# Cultural adaptation and intervention integrity: a response to Skärstrand, Sundell and Andréasson

**DOI:** 10.1093/eurpub/cku039

**Published:** 2014-04-09

**Authors:** Jeremy Segrott, Jo Holliday, Heather Rothwell, David Foxcroft, Simon Murphy, Jonathan Scourfield, Kerenza Hood, Laurence Moore

**Affiliations:** ^1^Centre for the Development and Evaluation of Complex Interventions for Public Health Improvement (DECIPHer), Cardiff School of Social Sciences, Cardiff University, Wales, UK, ^2^Faculty of Health and Life Sciences, Oxford Brookes University, Oxford, UK, ^3^Cardiff School of Social Sciences, Cardiff University, Wales, UK, ^4^South East Wales Trials Unit, School of Medicine, Cardiff University, Wales, UK and ^5^MRC/CSO Social and Public Health Sciences Unit, University of Glasgow, Glasgow, UK

A key issue in the cultural adaptation of interventions concerns the tension between modifying programmes to meet the needs of new populations and implementation settings, and the importance of retaining fidelity to the original intervention design.^1^ Skärstrand *et al.*’s recent randomized controlled trial (RCT) of the Swedish adaptation of Strengthening Families Programme (SFP) 10-14—a family-based substance misuse prevention intervention—found that it was ineffective in preventing drunkenness or drug or tobacco use.^2^ We are concerned that the null result could be interpreted as evidence that the SFP 10-14 is ineffective, whereas adaptations made to the intervention may have undermined intervention fidelity and theory by removing key family-based activities that play an important role in its hypothesized pathways to behaviour change.

The original American SFP 10-14^3^ (and its use in countries such as the UK and Poland^4^^,^^5^) comprises delivery to groups of 10–12 families, which parents and their child(ren) attend together. Parents and young people undertake separate activities in the first hour of each weekly session, and families are engaged in joint activities in the second hour to practise together the skills they have learnt. In many cases a meal is also offered to families at the halfway point of each session, providing an opportunity for social interaction within and between families. The focus on family level change, and the involvement of parents and children working together to develop new skills, thus differentiates SFP 10-14 from both traditional school-based health education (delivered only to pupils) and parenting (delivered only to parents) programmes.

Changes made to the SFP 10-14 during its adaptation for Sweden^6^ included: delivery of young people and parent sessions at different times (with the former during the school day and the latter in the evening); removal of the family hour in 6 of the 7 weeks of the programme sessions; providing the intervention to children whose parents/carers did not participate; delivery of the young people’s hour to whole classes of 25–30 young people, rather than groups of 10–12 participants; and inclusion of the booster programme sessions as a continuation of the 7-week programme (with no family session in 3 of the 4 weeks). The removal of the family sessions, and the decision to deliver the parent and young people’s sessions at different times, appear to have been driven primarily by local implementation needs and available resources (the cost of facilitators and the need to use school staff to help run the programme), rather than to optimize intervention fidelity or effectiveness.

As Stirman et al.^7^ argue, ‘While some [intervention] modifications might facilitate implementation and sustainability by improving the fit between the intervention and its target population or the context into which it is introduced, modifications may also erode treatment integrity’. The authors of the Swedish study conclude that there may be a number of potential reasons for their results showing the ineffectiveness of SFP 10-14, including the changes made to the programme during the cultural adaptation process, and/or contextual factors (e.g. lower rates of social disparities in Sweden creating ceiling effects). The authors suggest that they retained all the programme’s core content and components, but also state that ‘Possibly, the missing family parts are vital to the effectiveness of the programme’. In the logic model that we have developed as part of our RCT of SFP 10-14 in the UK^8^ (logic model available from the authors), key programme processes depend on the interaction between family members (e.g. the definition and articulation of family values)—underlining the importance of the family hour as an intervention component. The Swedish adaptation of SFP 10-14 may have covered all of the core programme *content*. But a key question is whether the changes in the learning environment in which it was delivered (removal of sessions in which parents and children worked together to practise skills, and the loss of interaction between families during programme activities and meal breaks) may have diminished or interrupted key *processes* and causal pathways. As the authors indicate, the finding that the Swedish adaptation of SFP 10-14 was ineffective may be related to these changes, rather than being an inherent failure of programme theory, cultural inappropriateness, or the quality of delivery. It is also important to note that because many parents did not attend the programme at all (only 47% of those whose children received the programme attended at least one session), many young people whose outcomes were measured came from families who received neither the family nor the parents’ hours, a major difference from implementation and evaluation of the programme elsewhere.

Frameworks such as the Medical Research Council guidance on developing and evaluating complex interventions identify the importance of establishing ‘a theoretical understanding of the likely process of change’ (p. 981), and assessing feasibility, before deciding on the appropriateness of conducting an effectiveness trial.^9^ It follows that when the content or delivery of interventions is altered significantly, the extent to which these modifications fit with (or disrupt) existing programme theories of change and logic models should be assessed. This includes assessing the role played by, and the interaction between, different intervention components. Various methods exist to undertake exploratory work of this kind, such as multiphase optimization strategy, which proceeds by *screening* (identifying key intervention components), *refining* (optimizing the dose of each component) and *confirmation* (assessing the effectiveness of the optimized intervention via an RCT), all of which pay close attention to programme theory.^10^ As Collins *et al.*^10^ suggest in relation to the multiphase optimization strategy framework, ‘… *a full confirmatory trial is mounted only when an optimized intervention has been reached*, and only when there is sufficient potential for efficacy (or effectiveness), based on the information gathered in the screening and refining phases’ [emphasis in original] (p. 72). It is not clear whether these preparatory stages were followed in the adaptation of this intervention in Sweden.

Cultural adaptation for local contexts is important, but it needs to retain fidelity to intervention theory. Intervention integrity depends not only on whether key topics are delivered to participants, but also whether activities and processes that facilitate hypothesized causal pathways to behaviour change take place. In the case of family-based interventions such as SFP 10-14, the form of delivery, the interaction within families and inter-family group dynamics all play a part. The significant changes made to the key components of the SFP 10-14 during its adaptation in Sweden raise questions about whether it is still appropriate to use the name Strengthening Families Programme 10-14, and if the results of the randomized trial might have been different had the original model been adhered to more closely.
